# Pathogenesis and Host Response in Syrian Hamsters following Intranasal Infection with Andes Virus

**DOI:** 10.1371/journal.ppat.1002426

**Published:** 2011-12-15

**Authors:** David Safronetz, Marko Zivcec, Rachel LaCasse, Friederike Feldmann, Rebecca Rosenke, Dan Long, Elaine Haddock, Douglas Brining, Donald Gardner, Heinz Feldmann, Hideki Ebihara

**Affiliations:** 1 Laboratory of Virology, Division of Intramural Research, National Institute of Allergy and Infectious Diseases, National Institutes of Health, Rocky Mountain Laboratories, Hamilton, Montana, United States of America; 2 Department of Medical Microbiology and Infectious Disease, University of Manitoba, Winnipeg, Manitoba, Canada; 3 Rocky Mountain Veterinary Branch, Division of Intramural Research, National Institute of Allergy and Infectious Diseases, National Institutes of Health, Rocky Mountain Laboratories, Hamilton, Montana, United States of America; 4 Office of Operations and Management, Division of Intramural Research, National Institute of Allergy and Infectious Diseases, National Institutes of Health, Rocky Mountain Laboratories, Montana, United States of America; Johns Hopkins University - Bloomberg School of Public Health, United States of America

## Abstract

Hantavirus pulmonary syndrome (HPS), also referred to as hantavirus cardiopulmonary syndrome (HCPS), is a rare but frequently fatal disease caused by New World hantaviruses. In humans HPS is associated with severe pulmonary edema and cardiogenic shock; however, the pathogenesis of this disease remains unclear largely due to a lack of suitable animal models for the study of disease progression. In this study we monitored clinical, virological, pathophysiological parameters and host immunological responses to decipher pathological factors and events in the lethal Syrian hamster model of HPS following intranasal inoculation of Andes virus. Transcriptional profiling of the host gene responses demonstrated a suppression of innate immune responses in most organs analyzed during the early stage of infection, except for in the lung which had low level activation of several pro-inflammatory genes. During this phase Andes virus established a systemic infection in hamsters, with viral antigen readily detectable in the endothelium of the majority of tissues analyzed by 7–8 days post-inoculation. Despite wide-spread infection, histological analysis confirmed pathological abnormalities were almost exclusively found in the lungs. Immediately preceding clinical signs of disease, intense activation of pro-inflammatory and Th1/Th2 responses were observed in the lungs as well as the heart, but not in peripheral organs, suggesting that localized immune-modulations by infection is paramount to pathogenesis. Throughout the course of infection a strong suppression of regulatory T-cell responses was noted and is hypothesized to be the basis of the aberrant immune activations. The unique and comprehensive monitoring of host immune responses to hantavirus infection increases our understanding of the immuno-pathogenesis of HPS and will facilitate the development of treatment strategies targeting deleterious host immunological responses.

## Author Summary

New World hantaviruses, including Andes virus (ANDV), are rodent-borne pathogens which are associated with hantavirus pulmonary syndrome (HPS) and case fatality rates up to 50%. The pathogenesis of HPS remains unclear; however, it is believed to involve a delicate balance of virus infection and deleterious immune-modulations. In this study, exploiting pathological and immunological approaches, we investigate the disease mechanisms of HPS caused by ANDV in the lethal Syrian hamster model. Despite systemic viral replication, pathological findings were almost exclusively observed in the lungs of infected hamsters. The most striking observations came from monitoring the host responses in infected and control hamsters. During early infection, innate responses in infected hamsters were down-regulated in all organs except lung, which demonstrated slight increases in pro-inflammatory transcripts. Immediately preceding the symptomatic phase, considerable increases in pro-inflammatory and T-cell activating cytokine responses were observed in lung and heart tissue, but not in other organs. Interestingly, expression of regulatory T-cell marker genes that might control deleterious inflammatory responses were suppressed throughout infection, suggesting a role in the immune-dysregulation during hantavirus infection. The results of this study provide an increased understanding of HPS pathogenesis and will aid in the development of novel therapeutic strategies to counter HPS.

## Introduction

Hantaviruses (family *Bunyaviridae*; genus *hantavirus*) are enveloped viruses which contain a tri-segmented, single-stranded, negative sense RNA genome encoding four proteins; the nucleoprotein, two glycoproteins (G_N_ and G_C_) and an RNA dependant RNA polymerase [1]. Currently, at least 22 hantavirus species have been described worldwide, half of which are pathogenic to humans [2]. Hemorrhagic fever with renal syndrome (HFRS) is responsible for greater than 200,000 cases annually across Europe and Asia, and is associated with mortality rates of up to 10% [3]. The more recently described hantavirus pulmonary syndrome (HPS) or hantavirus cardiopulmonary syndrome (HCPS) (here referred to as HPS) has a lower incidence of infection when compared to HFRS, however mortality rates range from 40–50% [3]. Cases of HPS have been diagnosed throughout the America's and are most commonly associated with infection of either Sin Nombre or Andes viruses (SNV and ANDV, respectively) [3], [4].

Clinically, HPS is defined as a febrile illness distinguished by diffuse, bilateral interstitial pulmonary infiltrates and compromised respiratory function which requires supplemental oxygen [5]. In humans, HPS is characterized by four phases of disease; febrile, cardiopulmonary, diuretic and convalescent [6]. The initial symptoms associated with the febrile phase are typically non-specific (i.e., fever, headache, general malaise and myalgia), however during the cardiopulmonary phase, HPS rapidly progresses from coughing and shortness of breath to shock and severe pulmonary edema requiring intubation and mechanical ventilation. The cardiopulmonary phase is the hallmark of HPS and is characterized by vascular leakage, which occurs primarily in the lungs, hypoxemia, and cardiac complications. Death can occur within 48 hours and in addition to respiratory failure, is due to shock and myocardial dysfunction. Patients who proceed to the diuretic phase rapidly improve, though the convalescent phase can last for months [6].

The pathogenesis of HPS remains unclear but likely involves a versatile balance of viral replication and immune modulation resulting in increased vascular permeability. *In vitro*, hantaviruses are capable of modulating the host cell immune response accomplishing both immune evasion in early stages of infection and immune activation later on [7]–[10]. These findings are supported by clinical findings of elevated pro- and anti-inflammatory Th1/Th2 cytokine responses detected in serum from HPS cases [11] and an increase in cytokine producing cells in lung sections prepared from fatal HPS cases [12]. In addition, unique excessive, hantavirus-specific T-cell responses have been documented in laboratory confirmed HPS cases and are positively correlated with disease severity [13]. Thus, an underlying immune dysregulation seems to play an important role in HPS disease progression, a hypothesis that has been difficult to prove *in vivo* without proper animal models.

Currently, only two disease models for hantaviruses have been described; infection of cynomolgus macaques (*Macaca fascicularis*) with wild-type Puumala virus, which mimics a mild form of HFRS known as nephropathia epidemica [14]–[16] and infection of Syrian hamsters (*Mesocricetus auratus*) with ANDV which results in a reproducible, lethal outcome with clinical signs similar to HPS in humans [17]–[19]. Interestingly, hantavirus infection in Syrian hamsters is not uniformly lethal. Aside from ANDV, only Maporal virus, a Venezuelan hantavirus not known to be pathogenic to humans, has been shown to cause HPS-like disease in hamsters, albeit with significantly reduced lethality [20]. Other etiological agents of HPS in humans, including SNV [19] and Choclo virus [21], are highly infectious but non-pathogenic in hamsters despite productive infection in endothelial cells of the lung microvasculature.

Here we examined HPS disease progression in Syrian hamsters following intranasal inoculation of a lethal dose of ANDV. In the present study we show that ANDV down regulates the host innate immune response early during infection allowing systemic spread of the virus. In addition, we found that immediately preceding clinical onset of disease, activation of innate and Th1/Th2 responses were observed, primarily in lung and heart, while T-regulatory cell responses were largely absent. Our results indicate that tissue specific ANDV immune-modulation is a key factor in HPS pathogenesis.

## Results

### Infection and disease progression

Inoculation of hamsters with 200 FFU of ANDV via the intranasal route resulted in 100% lethality occurring between 10 and 13 days post infection (p.i.). Following challenge, hamsters appeared normal with no obvious signs of adverse effects caused by the inoculation procedures. Overt signs of disease were not apparent in any animals until day 9 p.i., with individual hamsters succumbing to infection approximately 12–36 hours post onset of clinical signs. The majority of terminally ill hamsters presented with lethargy, hunched posture and dyspnea, while some also showed cyanosis and/or epistaxis.

**Figure 1:**
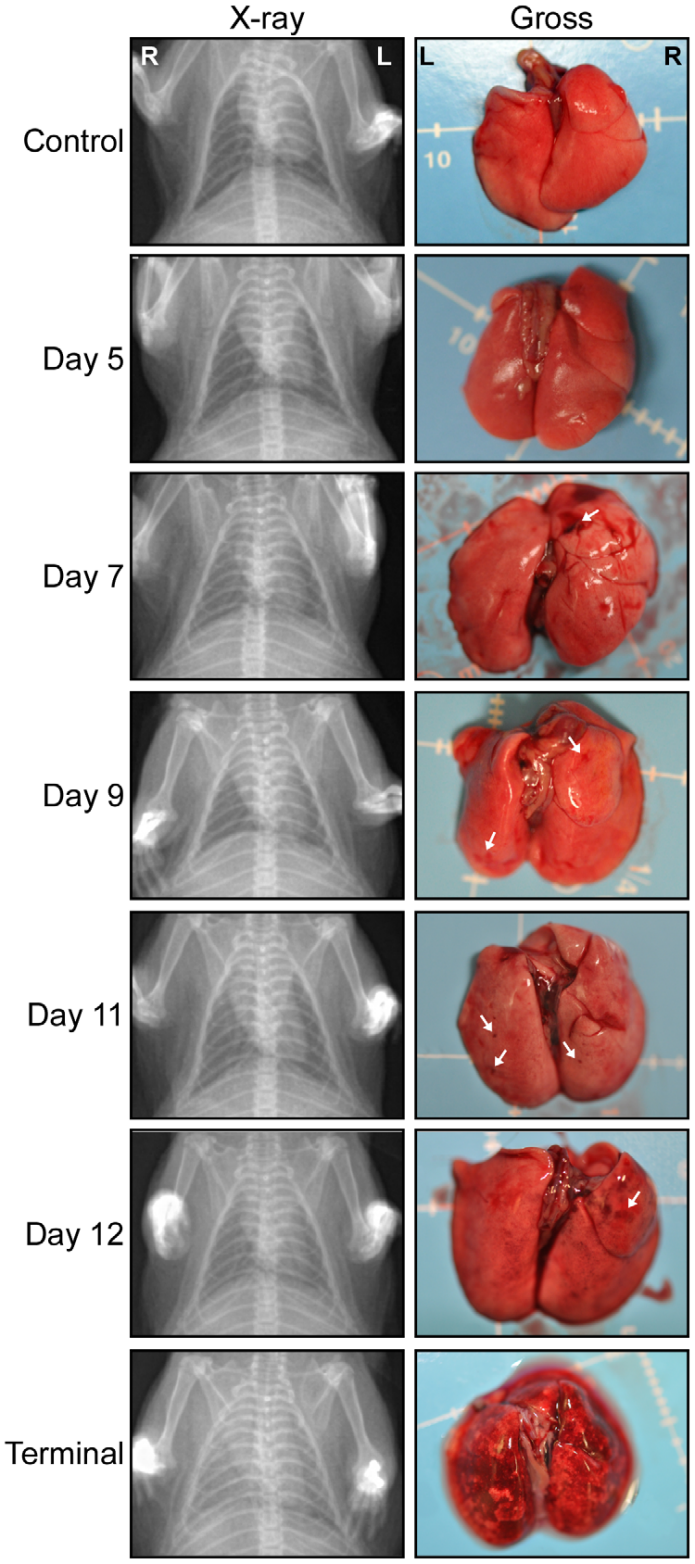
Radiographic imaging and gross lung pathology associated with HPS in hamsters. Hamsters were infected with 200 FFU of Andes virus as outlined in the materials and methods section. Beginning at 12 hours post-infection and continuing every 1–2 days, infected hamsters were euthanized and necropsied. Macroscopic evidence of pulmonary damage was first noted at day 7 post-infection (see arrows) with the frequency and severity of lesions increasing until the terminal phase of disease. Radiographic imaging (X-ray) demonstrated a progression of lung infiltrations beginning between days 7 and 9 post-infection. Shown are ventral dorsal chest radiographs and gross pathology from a representative hamster collected at the indicated time points.

**Figure 2:**
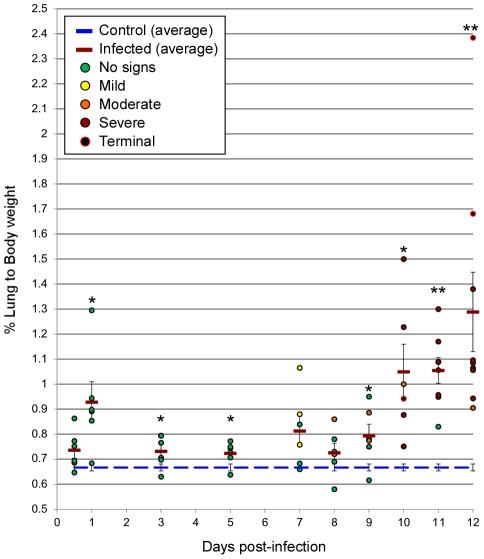
Lung weight to body weight ratios. Whole lungs were removed from Andes virus infected hamsters at indicated time points post-infection and lung to body weight ratios were determined. Individual dots represent data from a single hamster (expressed as a %) and are color coded to reflect the signs of infection/disease (as determined by clinical presentation and gross pathology of lungs, see inset for legend) of the specific animal. The blue dashed line represents the average lung to body weight ratio of control (uninfected) hamsters (n = 13) and the red dashed line represents the average ratios of infected hamsters at the indicated time point. Error bars represent the standard error of the mean. * Time point p<0.05; ** Time point p<0.01 (infected hamsters compared to controls).

Radiographic imaging was utilized to assess respiratory disease progression at regular intervals during the course of infection in a subset of 5 animals, as well as at the time of necropsy for all hamsters. Changes were first observed between days 7–9 p.i. with a minority of hamsters showing mild lung infiltration. After day 9 p.i., notable increases in opacity were observed throughout the lungs of most infected animals. At the time of clinical disease onset (days 9–11 p.i.), moderate to severe pleural effusions with diffuse pulmonary infiltrates were visible, followed by rapid pulmonary consolidation with the most drastic changes observed within the last 48 hours preceding death (Figure 1). Corroborating the radiographic observations, increases in the lung weight to body weight ratios were noted in infected animals, as compared to control (mock-infected) hamsters, especially after day 9 p.i. (Figure 2). An average of 3–5 mL of pleural fluid was excised from a subset of terminally ill hamsters.

### Virus detection

**Figure 3:**
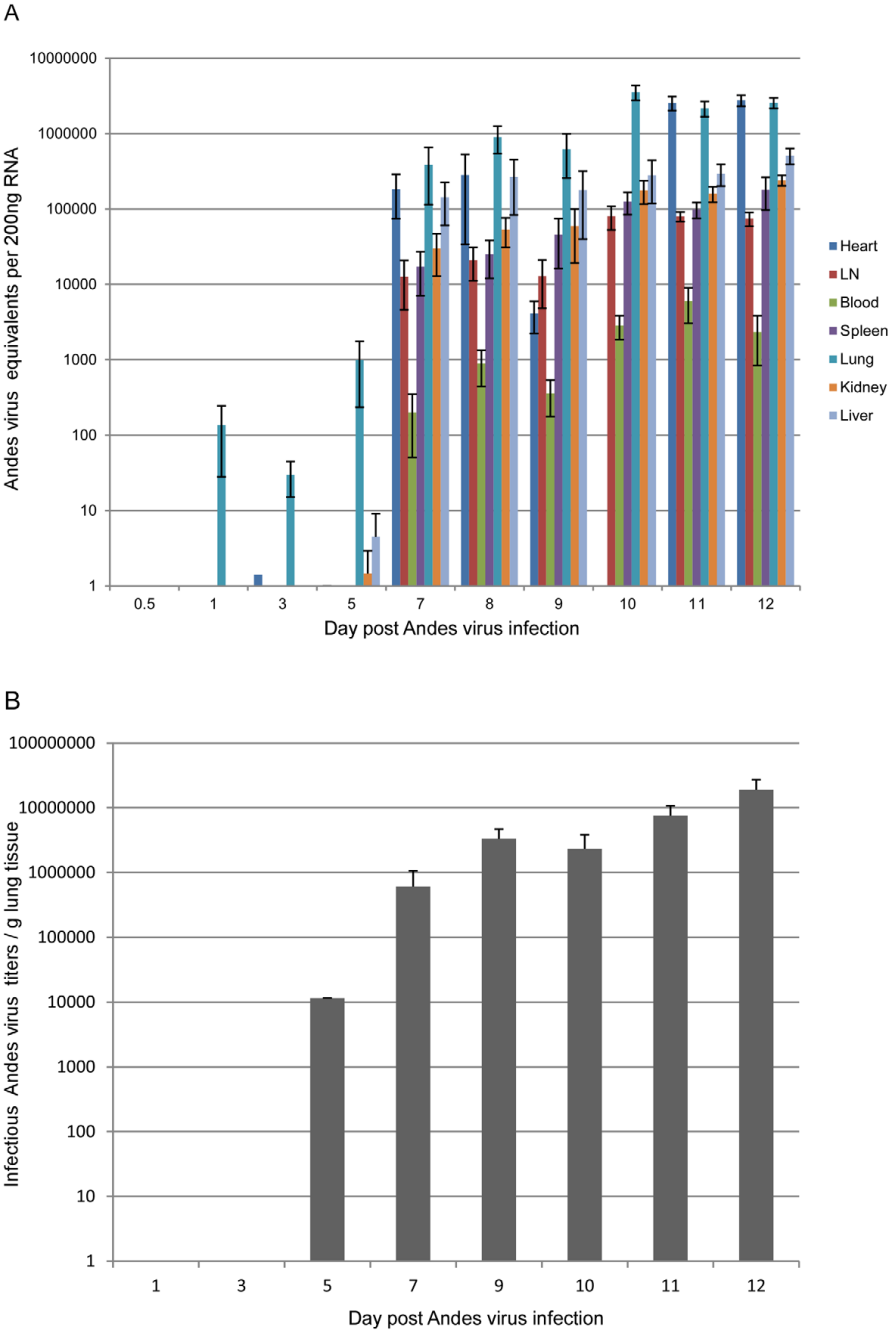
Andes virus organ titers. ANDV RNA was quantified in organs collected from infected hamsters at the indicated time points using primers and probe designed against the nucleoprotein coding region (A). Heart samples were not available for analysis at the day 10 time point due to insufficient levels of RNA. Error bars represent the standard error of the mean. LN, lymph nodes. Infectious ANDV titers were determined in lung samples from infected hamsters at the indicated time points using a focus-forming unit assay (B). Error bars represent the standard error of the mean.

Total RNA was extracted from tissue samples and tested for the presence of ANDV RNA as previously described [22]. ANDV RNA was detected in the lung of infected animals, as early as 24 hours p.i.. Between days 5–7 p.i., all tissues collected from infected animals tested positive for ANDV RNA, with peak titers detected in lung and heart from terminally ill hamsters (Figure 3A). Viral RNA was first noted in the blood of infected animals on day 7 p.i. and viremia remained relatively low with genome equivalents <10∧4 (Figure 3A). Infectious viral titers were determined in a subset of lung samples from infected hamsters and confirmed the qRT-PCR results (Figure 3B). At no time was ANDV specific RNA detected in tissues and blood from control hamsters.

**Figure 4:**
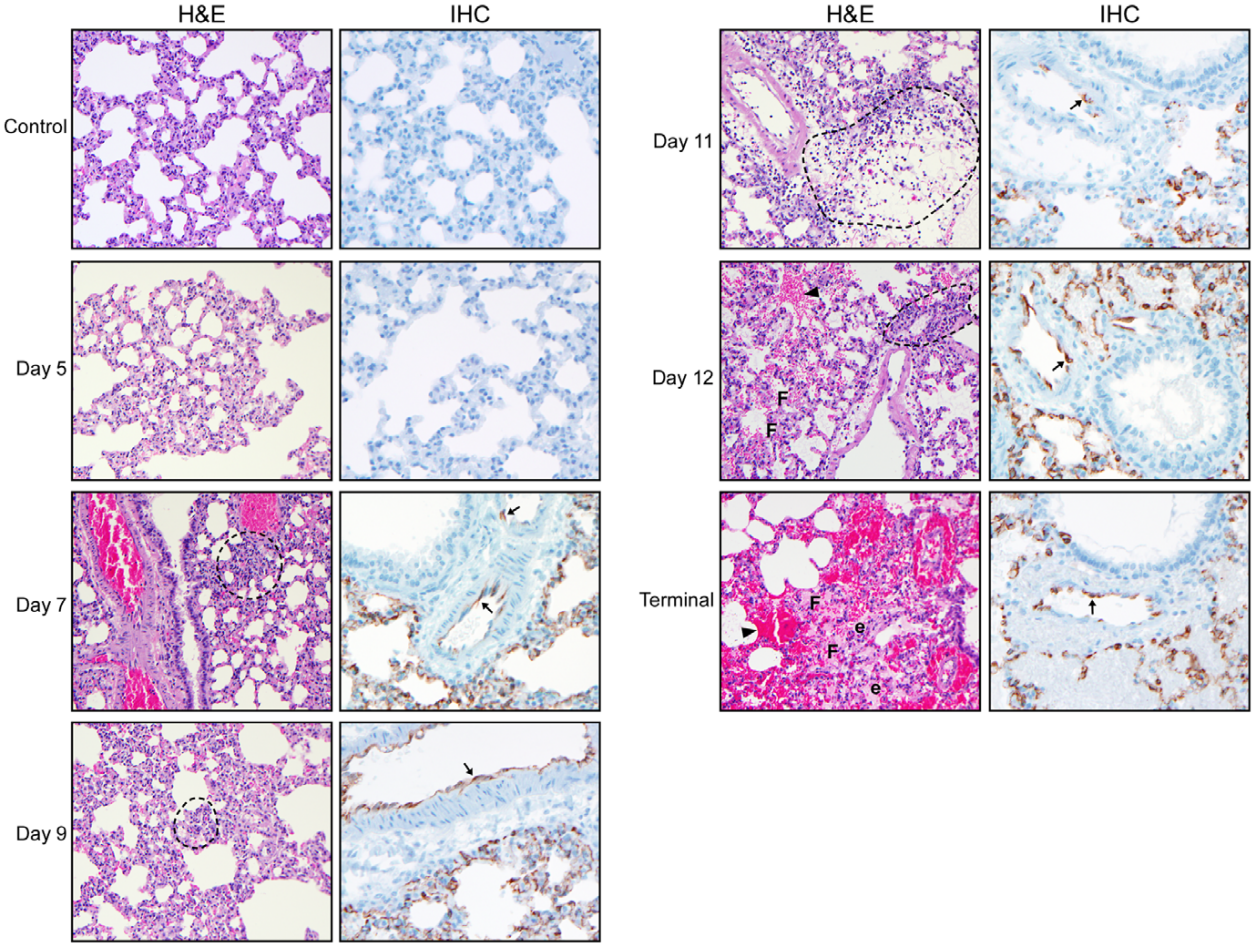
Histological examination of lungs from Andes infected hamsters. Hamsters were infected with 200 FFU of ANDV as outlined in the materials and methods section. Beginning at 12 hours post-infection and continuing every 1–2 days, infected hamsters were euthanized and necropsied. Shown are representative sections of lungs stained with hematoxylin and eosin (H&E, 200X magnification) or a monoclonal antibody targeting the ANDV nucleoprotein (IHC, 400X magnification) from a representative hamster collected at the indicated time points. Histological changes were first apparent at day 7 post-infection and included increased immune cell (predominantly macrophages, neutrophils and lymphocytes) infiltration (circled areas), hemorrhage (closed arrow heads), fibrin deposition (F) and edema (e). Diffuse staining of lung endothelium and alveolar septal cells (arrows) was also apparent beginning at 7 days post-infection. Note, samples shown correspond to the same hamsters in Figure 1.

**Figure 5:**
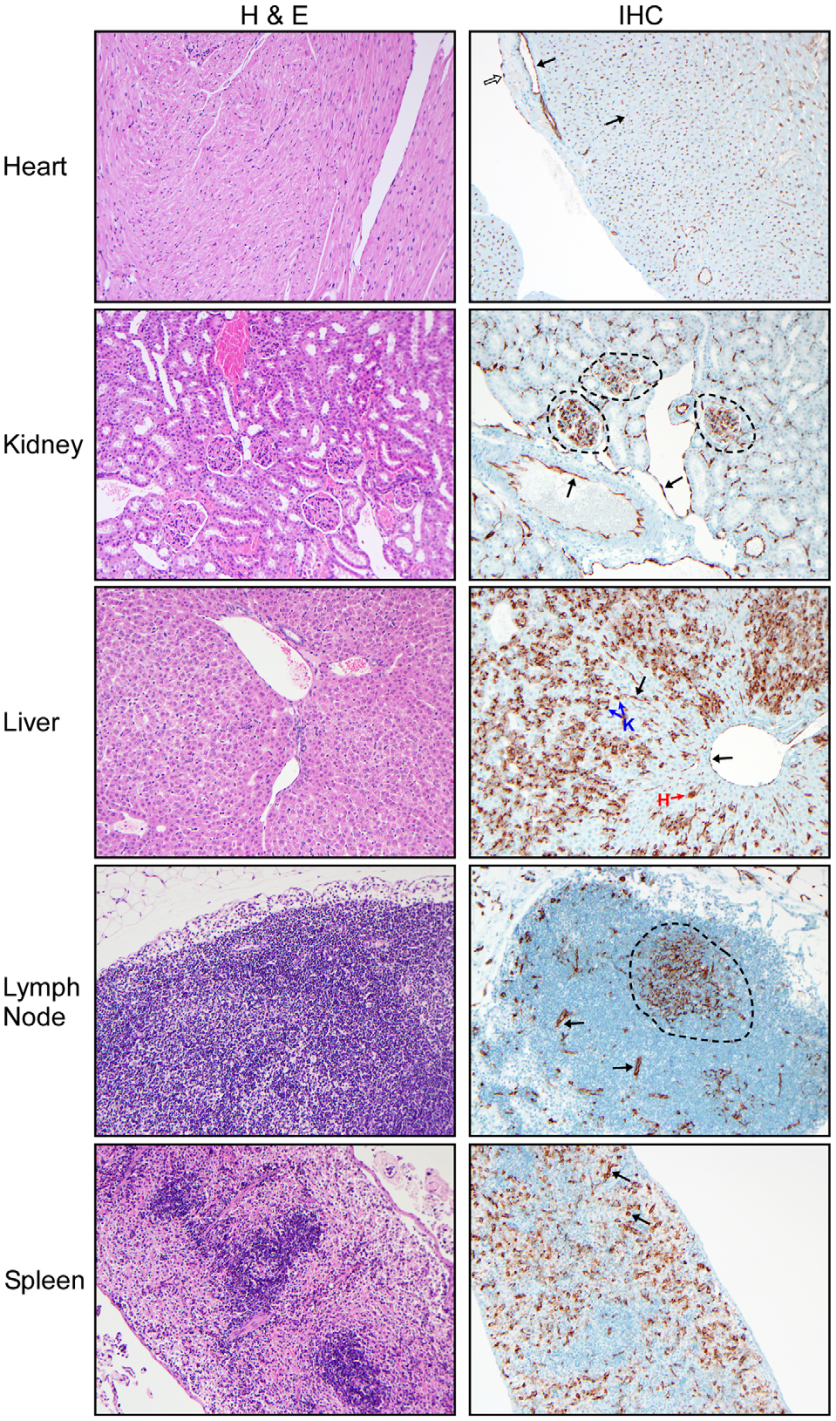
Histological analysis of peripheral organs from Andes virus infected hamsters. Representative samples of heart, kidney, liver, lymph nodes and spleen sections collected at day 12 post-infection from a terminally ill, ANDV infected hamster are shown (H&E 200X, anti-Andes virus nucleoprotein IHC 400X). Extensive viral antigen was noted in endothelial cells of all tissues analyzed (black arrow), including endocardial endothelium in heart sections (open arrow) and glomerular tuft endothelium in kidney sections (circled areas) as well as hepatocytes and Kupffer cells in liver sections (denoted with an H and K, respectively) and dendritic cells and macrophages in lymph nodes (circled area). Despite the high frequency of antigen positivity in peripheral tissue samples, histological abnormalities were infrequent and largely unremarkable in these organs.

**Figure 6:**
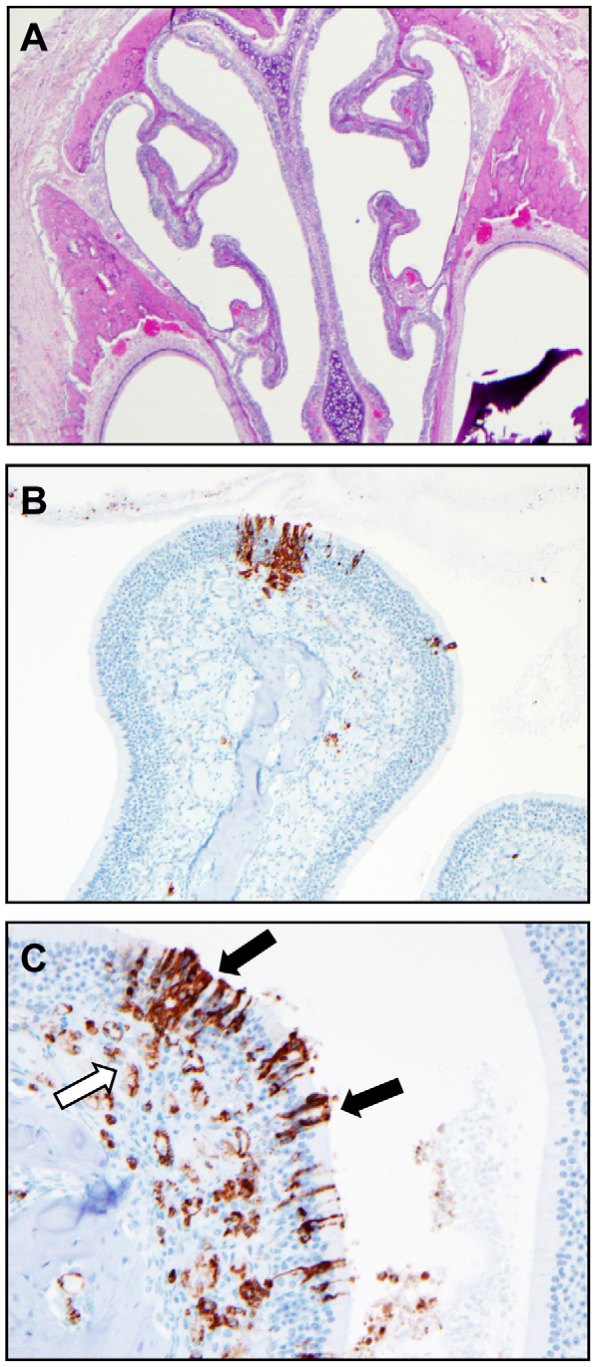
Histological analysis of the upper respiratory tract of Andes virus infected hamsters. At the conclusion of each necropsy, hamsters were decapitated and the head was skinned, fixed in 10% neutral buffered formalin, decalcified and sectioned for histological analysis of the upper respiratory tract. Throughout the course of the study no discernable virus induced pathology was noted in the upper respiratory tract of infected hamsters. (A, 40X magnification of upper respiratory tract of infected hamsters collected at day 10 post-infection). Immunohistochemistry demonstrates the presence of ANDV infected olfactory epithelial cells (black arrow) and endothelium (open arrow) in the upper respiratory tract of hamsters at days 10 (B, 200X magnification) and 12 (C, 400X magnification) p.i.

Immunohistochemistry (IHC) was accomplished using a monoclonal antibody generated against the ANDV nucleoprotein. Viral antigen was first observed at day 7 p.i. in multiple organs and cell types including, the lung vascular endothelium and alveolar septal lining cells (Figure 4), the endothelium of kidney (glomeruli and intertubular capillaries), trachea and heart (predominantly myocardial endothelium and, rarely, endocardial endothelium) as well as multifocal staining in the liver in hepatocytes and sinusoidal lining cells (most likely Kupffer cells and endothelial cells). In the spleen, antigen-positive cells were noted primarily in the red pulp and occasionally in cells with macrophage morphology, and the cervical lymph nodes, primarily in lymphoid follicles and endothelial cells. Representative sections of liver, kidney, spleen, heart and lymph node from a terminally ill hamster are shown in Figure 5. Similar patterns of antigen staining were observed in tissue sections examined from days 8–10 p.i., though intensity of staining increased in all sections, especially in lungs. Within the nasal cavity, antigen was detected in the endothelium and in olfactory epithelial cells after day 9 p.i. (Figure 6). During the terminal stages of disease (days 10–12 p.i.) there was consistent, strongly positive antigen staining in pulmonary alveolar septal cells and occasionally alveolar luminal cells most likely alveolar macrophages. There was no antigen staining in any tissues examined from control (uninfected) animals.

### Pathology and histopathology of ANDV infection

Gross pathological abnormalities were consistently noted in the lungs of infected animals. Beginning at day 7 p.i., macroscopic lesions were observed in the lungs of 3 of 6 infected animals. The frequency and severity of the affected areas increased until the terminal stage of disease at which point lesions covered ≥80% of all lung lobes and were hemorrhagic in appearance (Figure 1). Macroscopic evaluation of other tissues (trachea, liver, spleen, heart, kidney, digestive tract and cervical lymph nodes) was largely unremarkable with the exception of moderate hemorrhages noted in the cervical lymph nodes of approximately 50% of hamsters necropsied on days 11 and 12 p.i..

Similar to the gross pathology observed at necropsy, microscopic abnormalities were predominantly observed in the lungs of infected hamsters and consisted of interstitial pneumonitis and perivascular pulmonary inflammation with increased numbers of macrophages and neutrophils, expanding interstitial spaces, alveolar and perivascular edema, hemorrhage and fibrin deposition (Figure 4). The severity of lung pathology was rated as mild (severity scores 1–2) in specimens collected at 12 hours through day 5 p.i., with only minimally increased neutrophils and macrophages noted within alveolar septae and small foci within alveoli and bronchials. Between days 7 through 9 p.i., lung pathology was rates as mild to moderate (severity scores 2–3) with notable increases in macrophages and to a lesser extent neutrophils observed expanding the interstitial spaces. Lung specimens collected from days 10 and 11 were rated as moderate to severe (severity scores 3–4), and those from day 12 severe (severity score 4) with interstitial inflammation characterized by accumulations of macrophages, lymphocytes, edema, fibrin and hemorrhage. A definition of scoring parameters can be found in the materials and methods section. Throughout the study, histological changes in all lung lobes from individual infected animals sampled at all time points were consistent and uniform.

At no time point were any histological abnormalities observed in the heart, spleen, kidney (Figure 5), nasal tract (Figure 6), trachea, stomach or gastrointestinal tract (data not shown). Mild lymphocyte follicular and plasma cell hyperplasia were frequently noted in lymph nodes along with minimally increased neutrophils and eosinophils and interpreted as reactive lymph nodes and nonspecific changes (Figure 5). Histologic changes in the liver were infrequent and unremarkable until the terminal stages of infection (days 10–12 p.i.) with hepatocyte apoptosis and minimal lymphohistiocytic inflammation observed in a minority of animals (Figure 5). Minimal infiltrates of lymphocytes and macrophages in the meninges and surrounding blood vessels in the parenchyma (encephalitis) as well as mild hemorrhage were occasionally noted in the olfactory bulbs of the brain at days 10–12 p.i..

### Hematology, coagulopathy and blood clinical chemistries

Pronounced leukocytosis, mainly consisting of neutrophilia and lymphocytosis, was observed in infected hamsters as early as day 3 p.i. and continuing throughout the terminal stages of disease (data not shown). Clinical chemistries were mostly unremarkable in infected hamsters and differences in platelet counts were not observed. A slight (average of 5%) increase in hematocrit was documented, though only at days 11–12 p.i.. Examination of coagulation parameters (aPTT, PT, TT, Protein S, Protein C and fibrinogen concentrations) demonstrated a moderate increase in PT and aPTT and decrease in plasma fibrinogen levels which corresponded to the clinical phase of disease in hamsters (Table 1). Protein S activity fluctuated throughout the course of infection, with decreased activity noted beginning at day 3 p.i.. The remaining parameters showed minimal or no changes in infected hamsters as compared to the controls.

**Table 1:** Summary of coagulation parameters monitored in control and Andes infected hamsters.

Group	aPTT (s, ±SEM)	PT (s, ±SEM)	Fibrinogen (mg/dL, ±SEM)	Thrombin time (s, ±SEM)	Protein C* (%)	Protein S* (%)
Control	21.07±0.44	10.80±0.45	313.11±12.14	30.47±1.02	100	100
Day 1	20.05±0.99	9.75±0.15	347.13±22.74	26.65±0.68	100	100
Day 3	21.13±0.57	9.53±0.55	320.14±29.80	25.08±1.25	100	61.1
Day 5	21.55±0.20	9.43±0.44	265.20±37.56	27.92±1.26	92	74.4
Day 7	22.65±0.42	10.62±0.14	333.32±18.13	28.53±1.09	100	81.1
Day 8	22.02±1.18	11.50±0.18	324.52±10.88	26.42±1.42	100	91.1
Day 9	23.35±1.10	10.01±0.14	333.82±13.04	29.37±1.28	100	86.1
Day 10	24.98±1.53	12.14±1.36	290.33±59.70	32.57±3.87	100	91.7
Day 11	29.84±1.42*	10.66±0.49	262.72±46.97	33.53±4.73	100	60.6*
Day 12	43.10±3.46*	13.70±2.07*	148.90±47.02*	24.08±3.44	96	59.6*
p value	<0.001	0.0234	0.0115	-	-	0.0416

*Protein C and Protein S expressed as percent activity compared with control, uninfected hamsters.

### Host responses to infection

**Figure 7:**
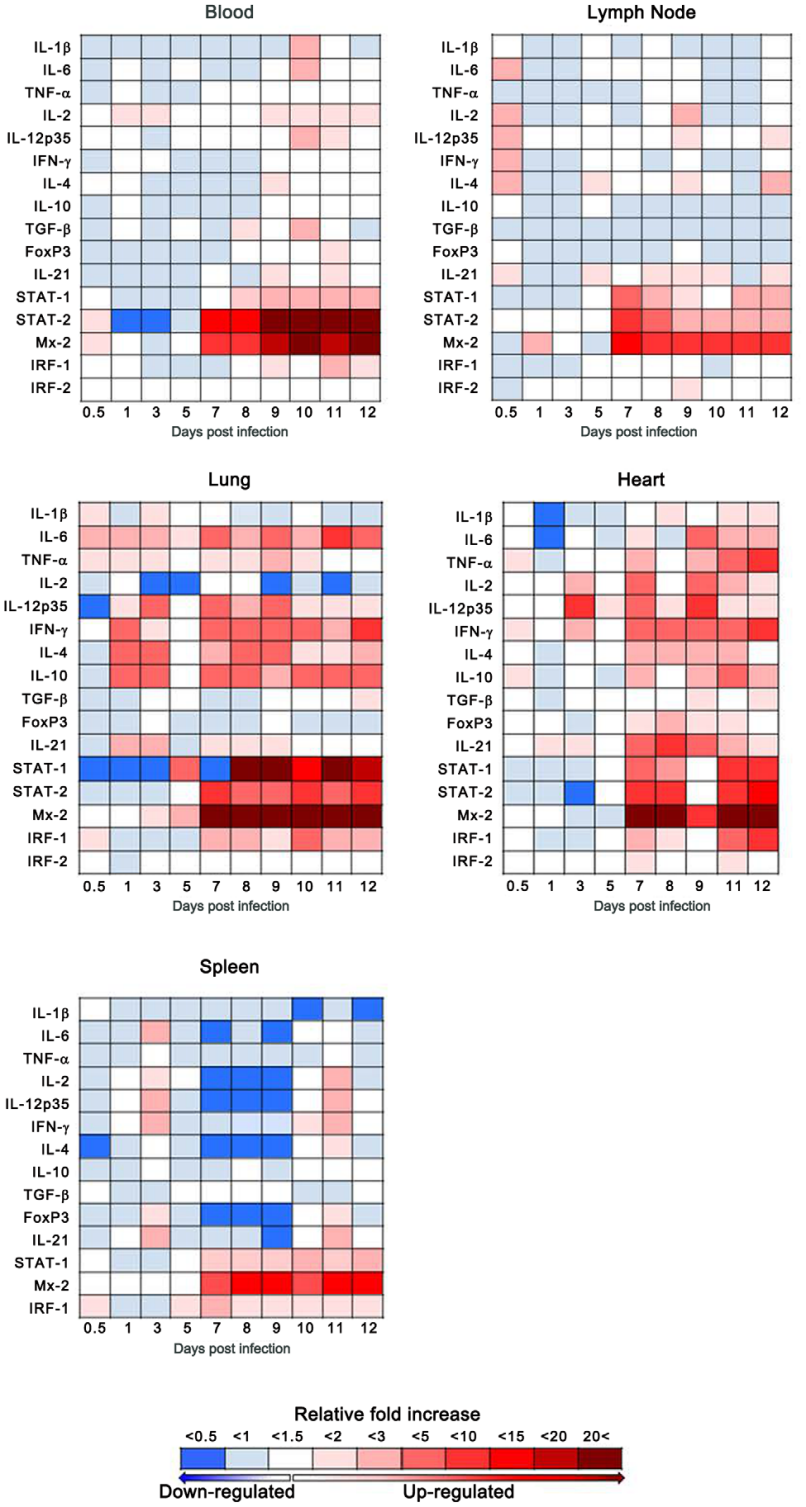
Host responses to Andes virus infection. Host responses to ANDV infection were monitored using recently developed, hamster specific, real-time RT-PCR assays. The average fold changes in lung, heart, spleen, cervical lymph nodes and blood samples from a minimum of 6 infected animals per time point are shown. Data collected is normalized uninfected animals. Day 10 heart samples were not tested due to insufficient RNA concentrations following extraction. Spleen samples were not tested for STAT-2 or IRF-2 for the same reason.

Host responses (including IL- 1β, 2, 4, 6, 10, 12p35 and 21, TNF α, IFN γ, TGF β, STAT1 and 2, IRF1 and 2, Mx2 and FoxP3) were monitored in lung, heart, spleen, cervical lymph nodes and blood samples from infected hamsters and compared to levels of control animals using recently developed, hamster specific real-time qRT-PCR assays (Figure 7, Figure S1) [23]. During the early stages of infection, no induction of the innate immune responses was observed in most tissues analyzed. The exception to this was slight increases in pro-inflammatory cytokine transcripts (IL-1β, IL-6, TNF α, IL-12p35, IFN γ and IL-21) in lung samples from infected hamsters. Beginning at day 7 p.i., transcripts increased for IRF-1, STAT-1, STAT-2 and particularly Mx2, most notable in lung, heart and blood. Following a similar pattern, activation of Th1 pro-inflammatory and Th2 anti-inflammatory transcriptional markers was observed in the heart with increased activation also observed in lung samples (compared to earlier time points). Most notable were IFN γ and IL-10 in both organs and TNF α and IL-6 in heart and lung, respectively. In comparison to most host responses monitored throughout the course of infection, expression of T-regulatory (Treg) cell marker genes (FoxP3 and TGF β) were relatively static or down regulated as shown in the lungs, with the exception of transient increases in heart and blood at days 7–9 corresponding to peak IL-21 expression in the heart.

Due to our small sampling size and the variability associated with differing responses to infection in outbred animals, a thorough statistical analysis of the temporal changes in cytokine transcripts within and between organs could not be accomplished. However, analyzing patterns of prolonged activation (i.e., ≥2 consecutive time points) revealed a significant amount of pro-inflammatory transcripts up-regulated in lungs during early infection (between days 1 and 7 p.i.), when compared to other organs examined (6 of 7 in lung, 0 of 7 for cervical lymph nodes and spleen each, p = 0.0210). At later time points, this trend continued in lung samples with 5 of 7 pro-inflammatory markers up-regulated compared 1 and 0 in spleen and lymph nodes respectively. Interestingly, during later disease (between days 7 and 12) a significant increase in the number of activated pro-inflammatory markers was observed in the heart when compared to lymph nodes and spleen (7 of 7 versus 0 and 1 respectively, p<0.01).

**Figure 8:**
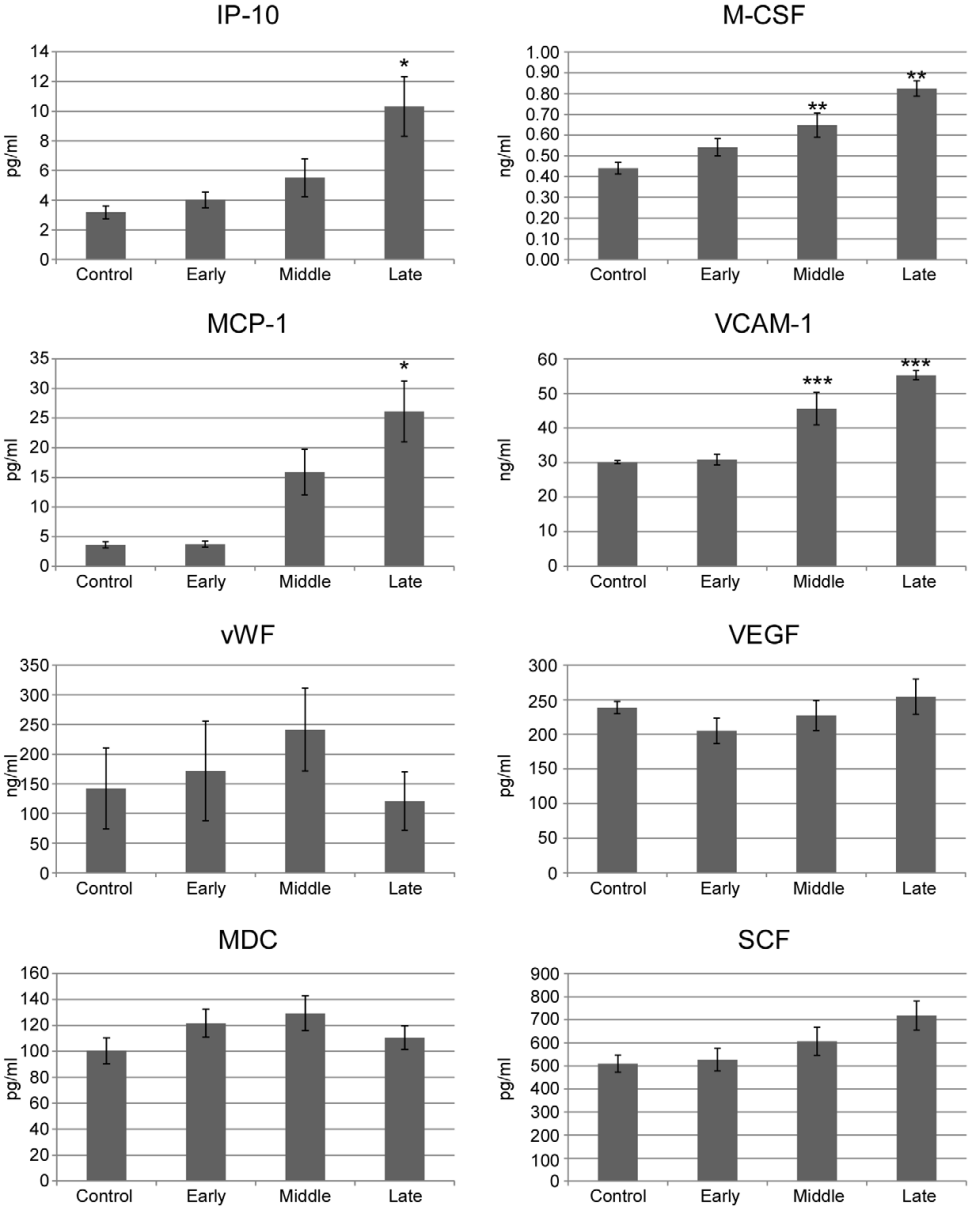
Analysis of plasma concentrations of inflammatory mediators. Plasma samples from control (n = 6) and Andes virus infected (n = 22) hamsters were tested for analytes using the RodentMAP version 2.0 platform. Samples from infected hamsters were divided into early (n = 6, collected on day 1 post-infection, p.i.), middle (n = 8, collected on days 7–8 p.i.), or late (n = 8, collected on days 11–12 p.i.) stages of disease. Shown are data from biomarkers that sufficiently cross-reactive with the mouse/rat specific assay. IP, inducible protein; M-CSF, macrophage-colony stimulating factor; MCP, monocyte chemoattractant protein; VCAM, vascular cell adhesion molecule; vWF, von Willebrand factor; VEGF, vascular endothelial cell growth factor; MDC, macrophage-derived chemokine; SCF, stem cell factor. * p<0.01, ** p<0.001; *** p<0.0001 (indicated time point to uninfected controls).

Host responses were also monitored in plasma samples from a subset of infected and control hamsters using a multiplex antigen capture-based assay as previously described [24]. In general, hamster cytokines could not be accurately quantified by the anti-mouse/rat cytokine-specific antibodies utilized in the 58-plex rodent assay, presumably due to lack of cross-reactivity, as demonstrated by values far below the least detectable dose and outside the range of the standard curve. However hamster chemokines, including IFN γ induced protein 10 (IP-10), macrophage-colony stimulating factor (M-CSF) and monocyte chemoattractant protein 1(MCP-1), were quantifiable with this assay (i.e., provided measurable values within the dynamic range of the assay) and demonstrated increased levels of detection throughout the course of infection (Figure 8). Other plasma factors, including vascular cell adhesion molecule 1(VCAM-1), von Willebrand factor (vWF), vascular endothelial cell growth factor (VEGF), macrophage-derived chemokine (MDC), stem cell factor (SCF), showed similar cross-reaction and demonstrated relative increases (VCAM-1) or no change (vWF, VEGF, MDC, SCF, Figure 8, data not shown), during the course of infection. Further validating these results, IHC performed with a polyclonal antibody specific for VEGF demonstrated no discernable differences in VEGF expression in lung samples collected from infected or control hamsters (data not shown).

## Discussion

The pathogenesis of HPS has basically evolved from studying a limited number of surviving and lethal HPS cases [6], [25], [26]. Efforts at modeling HPS disease in animals have largely failed, with the exception of the lethal ANDV model in Syrian hamsters [17]. The use of the hamster model has been hampered by the lack of species-specific reagents and therefore mainly focused on virology and histopathology [17]–[19]. Recently we have developed tools to follow certain hamster-specific immune responses upon stimulation at transcriptional level [23], which allowed us to perform a unique and comprehensive evaluation of the host immune responses in hamsters following intranasal ANDV infection, a route that mimics natural exposure of humans to hantaviruses.

### ANDV pathogenesis following intranasal infection of hamsters

Intranasal ANDV inoculation established a systemic infection in hamsters with viral specific RNA detectable in blood and all tissues analyzed from day 7 p.i. on (Figure 3A). The lung was the only organ that showed consistent virus replication starting at day 1 p.i. Despite systemic infection, histological changes were consistently observed only in the lungs of infected animals including pulmonary interstitial inflammation, edema, fibrin deposition and hemorrhages (Figures 1 and 4). Histological changes in other organs (heart, kidney, liver, cervical lymph nodes, spleen and trachea) were infrequent, minor, and only apparent in samples from terminal animals, making their contribution to disease development less likely. The lung, therefore is the predominant affected organ during infection and responsible for the clinical disease manifestation. However, mild to moderate subacute hepatitis and hepatocellular necrosis have been described in hamsters intramuscularly or intraperitoneally infected with ANDV [17, 19; own unpublished data] suggesting differences in disease development depending on the route of infection.

Previously, it has been reported that ANDV is capable of establishing a productive infection in primary respiratory epithelial cell cultures [27]. Here we assessed the role of the upper respiratory tract tissues for hantavirus replication following intranasal inoculation. Interestingly, sections of the nasal cavity were uniformly ANDV antigen negative until day 9 p.i., after which focal, patchy antigen staining of the epithelial cell lining, most notably in the olfactory epithelial cells, was observed in the absence of any discernable viral induced cellular damage (Figure 6). Despite an increase in antigen staining intensity of single infected cells over time, spread to surrounding epithelial cells was rare. This is in contrast to virus spread in the endothelium of other organs such as lung, kidney, heart and liver. These results suggest that epithelial cells of the upper respiratory tract are capable of supporting ANDV replication but are unlikely to play an important role in virus dissemination. However, we speculate that infection of epithelial cells later in infection might contribute to host to host transmission, a phenomenon that has only been described for ANDV infections in humans [28], [29].

The earliest signs of infection were manifest in hematology with pronounced increases of neutrophils and lymphocytes beginning at day 3 p.i. and continuing throughout the course of infection. Similar hematological changes have been described in HPS cases [30]. Examination of coagulation parameters indicated coagulation abnormalities with a weak tendency for hemorrhages. The most prominent observations were prolonged aPTT values and decreased plasma fibrinogen concentrations during the symptomatic phase (Table 1). Decreases in fibrinogen concentrations are associated with impairment of primary and secondary hemostasis, which results in increased clotting times as observed here. Similar findings have also been noted in Chilean HPS cases [30]. Interestingly, hemorrhagic manifestations were uniquely observed in South American HPS cases suggesting that ANDV possesses the potential to cause hemorrhages as supported by epistaxis in terminal ANDV infected hamsters.

Recently it was shown that ANDV infection of primary lung endothelial cells resulted in upregulation of VEGF and downregulation of vascular endothelial (VE)-cadherin, both of which are associated with increased vascular permeability [31]. While we were unable to monitor VE-cadherin, no differences in expression of VEGF in infected or control lungs were observed by IHC (data not shown) and plasma concentrations of VEGF did not differ between infected and control hamsters (Figure 8).

### ANDV down-regulates innate immune responses in hamsters early in infection

As known from other virulent RNA virus infections [32], [33], modulating the host cell immune response seems to be key for establishing hantavirus infections. *In vitro* studies have demonstrated that human pathogenic hantaviruses down regulate the innate immune response early after infection [8], [9]. Similarly, a recent study by Stoltz and colleagues demonstrated decreased serum concentrations of IFN-lambda in patients infected with hantaviruses, providing evidence of down regulation of innate responses *in vivo* [34]. In our study, a global suppression of innate immune-related responses, particularly for the type I interferon responses, was observed early after infection until about day 7 p.i. (Figure 7). The exception to this was low-level induction of pro-inflammatory responses in the lung. Subsequent up-regulation of innate immune responses correlated with both viremia and the appearance of viral antigen in the majority of organs. Despite continuing up-regulation of these responses ANDV continued to replicate, as demonstrated by increasing intensities of IHC staining and increasing RNA titers suggesting that following day 7 p.i., the immune response is overwhelmed and no longer capable of controlling ANDV replication.

### ANDV causes downregulation of Treg cell markers in hamsters

It is well established that downregulation of Treg cells results in disrupted immune homeostasis leading to immunopathology, while a vigorous Treg response can result in viral persistence [35], [36]. Recent studies have suggested an important role of Treg cells for hantavirus persistence in rodents [37]–[39] and disease development in HPS [11] and HFRS [40] patients. Schountz and colleagues demonstrated that deer mice persistently infected with SNV have a robust TGF β response and they speculated that Treg cells play an important role in limiting the immunopathology in infected deer mice [37]. Similar results have been documented in rats persistently infected with Seoul virus, an Old World hantavirus associated with HFRS in humans [38]. Conversely, decreased levels of TGF β associated with increased pro-inflammatory responses have been documented in HPS patients, supporting a deficient Treg response as a key factor in HPS immunopathogenesis [11]. Throughout the course of ANDV infection in hamsters, relatively static or decreased transcription levels of TGF β and FoxP3 were observed (Figure 7, Figure S1), supporting that ANDV is capable of modulating Treg cell responses likely contributing to immunopathogenesis of HPS. Although the target organ of HFRS differs from HPS, a recent study demonstrated that Treg responses in HFRS patients were negatively correlated with disease severity, suggesting a similar mechanism of pathogenesis [40].

### Organ-specific host responses to HPS

Pathology was focused in the lungs of ANDV infected hamsters which correlates well with the early and eventually strong immune response to infection in the lung and the primarily respiratory disease manifestation. Other organs are clearly infected, with evidence for ANDV replication in endothelial cells, but with the exception of the heart, measurable Th1/Th2 immune responses could not be detected in these organs (Figures 5 and 7). The mechanism responsible for the organ-specificity of the immune responses is unclear, however does not rely exclusively on viral replication. Supporting this, other HPS-causing hantaviruses have been shown to replicate in the lung microvasculature of hamsters without apparent disease manifestations [21]. Cells of the respiratory system appear to respond differently to ANDV infection. Interestingly, slight increases in a significant number of pro-inflammatory cytokine responses were observed in lungs early in ANDV infection (Figure 7). Early, low level, induction of inflammatory cytokines may prime the lung for the ensuing uncontrolled immune responses and subsequent immune-mediated pathogenesis, without negatively impacting viral replication. Interestingly, immediately prior to physical signs of disease, a significant amount of pro-inflammatory cytokine markers were detected in heart samples, suggesting cardiac activation occurs later in disease and may provide the final coup d'état.

Our present study strongly suggests that HPS can be categorized as a severe disease induced by organ-specific pathological changes associated with dysregulation of organ-specific host responses. Supporting this, a study by Mori and colleagues demonstrated an increase of cytokine producing lung cells in HPS cases, but not other organs [12]. Saggioro et al. described elevated levels of TNF α in the heart of HPS cases [41]. While neither of these studies analyzed both organs, heart and lung, from individual cases, the results support an organ-specific host response in HPS cases similar to that described here in ANDV infected hamsters. While we were unable to monitor circulating cytokine responses (lack of tools for hamsters), our results suggest the elevated levels of plasma cytokines observed in HPS cases originate from activated lymphocytes, endothelial cells and/or macrophages from lungs and heart.

The role of TNF α in the pathogenesis of HPS and particularly for increased vascular permeability is controversial [42], [43]. Our study showed weak to moderate levels of TNF α in lung and heart of ANDV infected animals. Saggioro et al. detected elevated levels TNF α in heart tissue from HPS cases [41] and suggested its role in myocardial depression and shock. Signs of cardiogenic shock have also been reported in the terminal stages of HPS disease in hamsters [18]. While we were unable to monitor cardiogenic shock, increased cytokine levels in heart and lungs from infected hamsters (Figure 7, Figure S1) and humans [11], [41] including elevated levels of TNF α and IL-6, which both suppress myocardial function [44], [45], and IL-12, which has been associated with myocarditis in mice [46] support cardiac depression in HPS. Curiously, our findings of increased IL-12 expression in heart samples suggest a Th1 response yet no evidence of Th1 type pathology was noted in heart samples, possibly due to a regulatory effect of IFN γ [46]. Despite the lack of apparent viral induced pathology in heart tissue of ANDV infected hamsters, expression of certain cytokines with impact on myocardial function support the role for cardiac involvement in HPS and lends support to the alternative designation HCPS [3].

Of note is the increased IL-21 expression in heart tissue of ANDV infected hamsters. IL-21 plays a critical role in the differentiation of Th17 cells, a newly recognized subset of T cells with opposite function to Treg cells and involvement in autoimmune processes [47], [48]. Previously, it has been speculated that an unfavorable imbalance of immuno-regulatory Treg cells and immuno-pathogenic Th17 cells contribute to pathogenesis of a diverse group of immune-mediated diseases, including inflammatory disease and acute heart failure [48]–[50], two syndromes associated with HPS. Thus future studies need to confirm a potential novel role of impaired Treg responses and Th17 differentiation for the immunopathogenesis of HPS.

The dogma of T cell mediated immunopathogenesis of hantavirus infections was recently challenged by Hammerbeck and Hooper [51]. Using the hamster model of HPS, the authors demonstrated increases in activated T cells in lung and spleen samples from ANDV infected hamsters which correlated with the onset of disease and subsequent death. Interestingly, they also found that HPS disease progression and lethal outcome in hamsters depleted for CD4+ and/or CD8+ T cells was indistinguishable from that in undepleted animals, suggesting that T cells are not critical in HPS disease development [51].

The timing of the increases in activated T cells noted by Hammerbeck and Hooper in undepleted hamsters appears to correlate with the onset of the aberrant immune responses observed in our study (Figure 7) [51]. Interestingly, although increases in activated T cells were noted in both lung and spleen [51], in our study the aberrant immune responses were noted in the lung, but not the spleen of infected animals. While we are unable to define the cell types responsible for increased cytokine production, combined with the results of Hammerbeck and Hooper, it is reasonable to believe that the tissue specific, abnormal immune response in ANDV infected hamsters is not exclusively due to T cells. Other cell types, for example monocytes, dendritic cells or alveolar macrophages, may also play an important role. However, since T cell depletions were conducted at 6 to 8 days post-ANDV inoculation [51], the current data cannot rule out the effects of early activation of T cells and its effects on potential cross-talk with other cell types noted above, as well as the pulmonary endothelium. Furthermore, a recent review of the pathogenesis of HPS and HFRS suggests a more intricate balance of T cell involvement in which cellular responses that are too aggressive or too weak may similarly result in severe disease [52]. This may in part explain the results of the recent depletion study [51]. Nevertheless, the study by Hammerbeck and Hooper [51] along with our study, highlight the complexity of HPS pathogenesis and demonstrate the utility of the hamster model for elucidating mechanisms of disease development.

### Systemic mediators of infection

Attempts to quantify plasma cytokine responses in hamsters using reagents developed for other rodent species were largely unproductive. Most likely this is due to a lack of cross-reaction, which has been shown previously in Pichinde infected hamsters [24]. Attempts to detect hamster cytokines using rodent specific ELISA and Luminex panels were also unproductive [23, own unpublished data]. Despite this, hamster chemokines were quantifiable with the Rodent-MAP platform and several demonstrated increased levels during the course of ANDV infection. Of note were increases in immunoattractant proteins including MCP-1, M-CSF and IP-10, which has been previously shown to be induced following hantavirus infection in primary endothelial cell culture [53]. Combined these proteins likely provide the chemotaxis for immune cell infiltration observed in the lungs. Although we were unable to quantify hamster cytokines beyond the transcript level, upregulation of IP-10 and VCAM-1 indirectly support increased cytokine production since both are induced by cytokines including IFN γ and TNF α.

### Model of HPS pathogenesis in Syrian hamsters

In conclusion, following intranasal infection, a broad suppression of innate responses allows ANDV to establish an infection in the lower respiratory tract with subsequent systemic spread. Early measurable signs of infection are hematological abnormalities and pulmonary infiltration, primarily consisting of neutrophils and macrophages early, and macrophages and lymphocytes later in disease. A crucial time during infection lies around day 7 p.i., when initial immune suppression switches to immune activation which appears to be triggered through continuous suppression of host regulatory responses (i.e., Tregs), and uncontrolled viral replication. The terminal stage starts with the appearance of clinical signs around day 9 p.i. when radiographic changes can be observed. This period is short and animals succumb to infection within 36–48 hours, as has been described for human HPS cases. Virus dissemination seems to occur through viremia but the primary organ for virus replication and immune-associated damage through host responses is the lung followed by the heart. Thus, pathogenesis seems to be a result of a combination of virus replication with an early suppression of the host innate immune responses followed by a virus-triggered organ-specific deleterious host immune response resulting in pulmonary damage and cardiac suppression leading to respiratory distress.

## Materials and Methods

### Ethics statement

All animal experiments were approved by the Institutional Animal Care and Use Committee of the Rocky Mountain Laboratories (ASP #2009-43), and performed following the guidelines of the Association for Assessment and Accreditation of Laboratory Animal Care, International (AAALAC) by certified staff in an AAALAC approved facility.

### Biosafety and containment

All work with infected hamsters and potentially infectious materials derived from hamsters was conducted in the Biosafety Level 4 facility at the Rocky Mountain Laboratories, Division of Intramural Research, National Institute of Allergy and Infectious Diseases, National Institutes of Health. Sample inactivation and removal was performed according to standard operating protocols approved by the local Institutional Biosafety Committee.

### Inoculation of hamsters

ANDV, strain Chile 9717869 [54], was propagated and titered as previously described [22]. Syrian hamsters (female, aged 4–6 weeks) were inoculated with 200 focus forming units (FFU) of ANDV (100×LD_50_), a dose previously determined to be uniformly lethal in this model, diluted in sterile DMEM and administered through the intranasal route with 50 µl of virus suspension delivered per nare using a sterile p200 pipette. Control animals received an equivalent volume of sterile DMEM alone.

### Sample collection

At 12 hours and 1, 3, 5, 7, 8, 9, 10, 11 and 12 days p.i., one control and six infected hamsters were anesthetized via inhalational isoflurane and chest radiographs (ventral dorsal, right and left lateral) were taken using a portable digital radiography unit with a flat panel digital detector (TruDR, Sound-Eklin, Carlsbad, CA) and veterinary specific software (VET-PACS, London, United Kingdom). Hamsters were weighed, bled (EDTA and heparin treated 1.8 mL vacutainer tubes) and exsanguinated via cardiac puncture. Necropsies were performed to collect trachea, lung, liver, spleen, heart, kidney, digestive tract (stomach and intestines) and cervical lymph nodes. Lungs were removed, photographed, weighed, and a representative sample was taken from each lobe for histopathology (see below). In addition, a piece of the left lung was processed for RT-PCR as outlined below. Organ samples were processed for molecular analysis, histopathology and IHC. For molecular analysis, pieces of tissue (approximately 100 mg) were immersed in 1 mL of RNAlater (Qiagen, Valencia, CA) for overnight at 4°C, removed and stored at −80°C until RNA extraction. The remainders of each organ were fixed in 10% neutral buffered formalin for seven days at 4°C and processed as outlined below for histopathology and IHC. The head was skinned and fixed in 10% neutral buffered formalin for histological analysis of the upper respiratory tract. Lung weight to body weight ratios were calculated and pairwise comparisons of data from control and infected hamsters performed at each interval using t-tests.

### Clinical score

Hamsters were assigned a clinical score based on signs of infection/disease as determined by clinical presentation and gross pathology of lungs as follows: no signs  =  indistinguishable from controls; mild  =  hamsters appeared lethargic, lesions on single lung lobes; moderate  =  onset of breathing abnormalities (rapid shallow breathes), lesions apparent on multiple lung lobes; severe  =  breathing distress, multi-focal lesions of increased size on all lung lobes; terminal  =  moribund, epistasis, lungs hemorrhagic in appearance.

### Time-course radiographs

Disease progression was monitored in a subset of five ANDV infected hamsters with chest radiographs taken at one to two day intervals until day 7 p.i. and twice daily (approximately 12 hours apart) from day 7 p.i. until the point of euthanasia.

### Hematology and blood chemistry

Blood chemistry was monitored in heparinized blood using a portable iSTAT instrument (Abbott Point of Care, Princeton, NJ) with EC8+ cartridges measuring Na, K, Cl, TCO_2_, anion gap, glucose, urea nitrogen, hematocrit, hemoglobin, pH, pCO_2_, pO_2_, TCO_2_, HCO_3_, and base excess. Hematology, including white blood cell count, lymphocyte, platelet, reticulocyte and red blood cell counts, hematocrit values, mean cell volume, mean corpuscular volume, and mean corpuscular hemoglobin concentrations, was performed on EDTA blood using a Hemavet 950FS (Drew Scientific, Waterbury, CT).

### Coagulation testing

Due to the volume of plasma required for the coagulation examination, a second group of one control and six infected hamsters were exsanguinated via cardiac puncture at days 1, 3, 5, 7, 8, 9, 10, 11 and 12 p.i. with blood collected into 1.8 mL citrate vacutainers. Plasma was tested for coagulation parameters, including activated partial thromboplastin time (aPTT), prothrombin time (PT), thrombin time (TT), fibrinogen concentration, and Protein S and Protein C activity, on a STart4 instrument using the PTT Automate, STA Neoplastine CI plus, STA Thrombin, Fibri-Prest automate, STA Staclot Protein S and Protein C kits, respectively (all from Diagnostica Stago, Parsippany, NJ). Data was analyzed using a one-way analysis of variance (ANOVA) with Tukey-Kramer multiple comparison post-test comparing values from infected hamsters to uninfected controls.

### Histopathology and IHC

Formalin fixed tissues were processed and embedded in paraffin according to standard procedures. For sectioning of the nasal tract, the mandible and any extraneous muscle was removed from formalin fixed skulls to expose the cranium. Skulls were rinsed in running tap water, placed in a decalcifying solution consisting of 20% EDTA in sucrose (Newcomer Supply, Middleton WI) and allowed to sit at room temperature for 3–5 weeks, with 2–3 changes of decalcification solution over that period. Following decalcification, skulls were processed and embedded in paraffin.

Thin (5 µm) sections were cut and stained with hematoxylin and eosin or tested for the presence of viral antigen by IHC using a monoclonal antibody generated against the ANDV nucleoprotein (1:500 dilution, clone 1A8F6, Austral, San Ramon, CA). IHC was accomplished on a Discovery XT instrument (Ventana Medical Systems, Tucson, AZ) using a biotinylated goat anti-mouse (1:250 dilution, BioGenex, San Ramon, CA) secondary antibody and DAB Map kit. Following immunological staining, slides were counterstained with hematoxylin, dehydrated, cleared in xylene, and coverslipped. In addition, lung sections were stained for vascular endothelial growth factor (VEGF, 1:100 dilution, sc-507, Santa Cruz Biotechnology, Santa Cruz, CA) using a cross-reactive polyclonal antibody generated in rabbits essentially as outlined above with an alkaline phosphatase conjugated secondary antibody (Ventana Medical Systems).

Slides were evaluated by a Veterinary Pathologist. The scoring for H and E stained lung specimens were as follows: 1  =  Minimally increased numbers of inflammatory cells within alveolar septae without widening of the septae; 2  =  Mildly increased numbers of inflammatory cells within septae and mild expansion, or thickening, of septal walls and occasional extension of inflammation into the lumen of alveoli and bronchioles; 3  =  Interstitial inflammation (alveolar septae and perivascular, peribronchiolar and peribronchial) and inflammation within alveoli and larger airways (inflammatory cells, fibrin, hemorrhage). Inflammation may be extensive and involve large areas of the tissue section; 4  =  Changes are as described for #3; however, extent of lesions is greater and involves most of the tissue section.

### Transcriptional profiling of host response and virus replication

RNA was extracted from solid tissues (30 mg pieces) and blood samples using RNeasy or QIAamp viral RNA kits (Qiagen), respectively, according to manufacturers' instructions. Immediately after extraction, RNA was quantified on a nanodrop 8000 spectrophotometer (Thermo Scientific, Wilmington, DE). Sample concentration was adjusted to 40 ng/µl and aliquots frozen at -80°C. Real-time quantitative (q) RT-PCR was performed on a rotor-gene 6000 instrument (Corbett Life Science, Sydney Australia) using QuantiFast probe reagents (Qiagen) and 200 ng of template RNA. The presence of ANDV RNA was quantified using a previously described nucleoprotein specific assay [22]. Host responses, including interleukin (IL) 1β, 2, 4, 6, 10, 12p35, and 21, tumor necrosis factor alpha (TNF α), interferon gamma (IFN γ), transforming growth factor beta (TGF β), forkhead box P3 (FoxP3), myxovirus resistance protein 2 (Mx2), interferon regulatory factor 1 (IRF1) and 2 (IRF2), and signal transducer and activator of transcription 1 (STAT1) and 2 (STAT2), were monitored in lung, spleen, heart, cervical lymph node and blood samples using recently developed Syrian hamster specific, real-time qRT-PCR assays using ribosomal protein L18 as an internal control [23]. The small group sizes used in these studies prevented a thorough statistical analysis of temporal transcriptional data; however, patterns of up-regulation of pro-inflammatory markers were analyzed using Chi square tests.

### Multiplex cytokine, chemokine, and plasma factor analysis

Plasma samples from a subset of infected (n = 22) and control (n = 6) hamsters were sent to Rules-Based Medicine, Inc (Austin, TX) for analysis of cytokine and chemokine concentrations using a 58-biomarker Multi-Analyte Profile (MAP) approach (RodentMAP version 2.0). Samples from infected animals were divided into three categories; early (n = 6, collected on day 1 p.i.), middle (n = 8, collected on days 7–8 p.i.), and late (n = 8, collected on days 11–12 p.i.) stages of disease. Although this platform has only been validated against mouse and rat samples, recently it was suggested to work for selected hamster biomarkers, especially chemokines [24]. Data was analyzed using a one-way ANOVA with Tukey-Kramer multiple comparison post-test.

### Virus titration

Infectious viral loads were measured in a subset of lung homogenates (10% [w/v]) from infected hamsters using a focus forming unit assay essentially as previously described [22].

### Accession numbers

GenBank accession numbers are as follows: IL-1β AB028497; IL-2 EU729351; IL-4 AF046213; IL-6 AB028635; IL-10 AF046210; IL-12p35 AB085791; IL-21 FJ664142; TNF α AF315292; IFN γ AF034482; TGF β AF046214; FoxP3 FJ 664148; Mx2 EU616539; IRF1 DQ092344; IRF2 AY714581; STAT1 DQ092343; STAT2 AB177399; ribosomal protein L18 DQ403027.

## Supporting Information

Figure S1 Host responses to Andes virus infection. Host responses to Andes virus infection were monitored using recently developed, hamster specific, real-time RT-PCR assays. Shown is the qRT-PCR data used to generate the heat maps in Figure 7. Error bars represent the standard error of the mean.
(TIFF)

## References

[1] Schmaljohn C, Nichol ST, Knipe DM, Howley PA (2007). Bunyaviridae.. Field's virology 5^th^ ed.

[2] Nichol ST, Elliott RM, Goldbach R, Plyusnin A, Schmaljohn CS, Fauquet CM, Mayo MA, Maniloff J, Desselberger U, Ball LA (2005). Bunyaviridae.. Virus Taxonomy, VIII^th^ Report of the International Committee on Taxonomy of Viruses.

[3] Jonsson CB, Figueiredo LTM, Vapalahti O (2010). A global perspective on hantavirus ecology, epidemiology and disease.. Clin Microbiol Rev.

[4] Khan AS, Young JC (2001). Hantavirus pulmonary syndrome: at the cross roads.. Curr Opin Infect Dis.

[5] CDC (2010). http://www.cdc.gov/ncphi/disss/nndss/casedef/hantaviruscurrent.htm.

[6] Enria DA, Briggiler AM, Pini N, Levis S (2001). Clinical manifestations of New World hantaviruses.. Curr Top Microbiol Immunol.

[7] Geimonen E, Neff S, Raymond T, Kocer SS, Gavrilovskaya (2002). Pathogenic and non-pathogenic hantaviruses differentially regulate endothelial cell responses.. Proc Natl Acad Sci U S A.

[8] Levine JR, Prescott J, Brown KS, Best SM, Ebihara H (2010). Antagonism of type I interferon responses by New World hantaviruses.. J Virol.

[9] Alff PJ, Gavrilovskaya IN, Gorbunova E, Endriss K, Chong Y (2006). The pathogenic NY-1 hantavirus G1 cytoplasmic tail inhibits RIG-I- and TBK-1-directed interferon responses.. J Virol.

[10] Spiropoulou CF, Albarino CG, Ksiazek TG, Rollin PE (2007). Andes and Prospect Hill hantaviruses differ in early induction of interferon although both can downregulate interferon signalling.. J Virol.

[11] Borges AA, Campos GM, Moreli ML, Souza RLM, Saggioro FP (2008). Role of Th1 and Th2 cytokines on pathogenesis and prognosis of hantavirus cardiopulmonary syndrome.. Microbe Infect.

[12] Mori M, Rothman AL, Kurane I, Montoya JM, Nolte KB (1999). High levels of cytokine-producing cells in the lung tissues of patients with fatal hantavirus pulmonary syndrome.. J Infect Dis.

[13] Kilpatrick ED, Terajima M, Koster FT, Catalina MD, Cruz J (2004). Role of specific CD8+ T cells in the severity of a fulminant zoonotic viral hemorrhagic fever, hantavirus pulmonary syndrome.. J Immunol.

[14] Groen J, Gerding M, Koeman JP, Roholl PJ, van Amerongen G (1995). A macaque model for hantavirus infection.. J Infect Dis.

[15] Klingstrom J, Plyusnin A, Vaheri A, Lundkvist A (2002). Wild-type Puumala hantavirus infection induces cytokines, C-reactive protein, creatinine, and nitric oxide in cynomolgus macaques.. J Virol.

[16] Sironen T, Klingstrom J, Vaheri A, Andersson LC, Lundkvist A (2008). Pathology of Puumala hantavirus infection in macaques.. PLoS One.

[17] Hooper JW, Larsen T, Custer DM, Schmaljohn CS (2001). A lethal disease model for hantavirus pulmonary syndrome.. Virology.

[18] Campen MJ, Milazzo ML, Fulhorst CF, Obot Akata CJ, Koster F (2006). Characterization of shock in a hamster model of hantavirus infection.. Virology.

[19] Wahl-Jensen V, Chapman J, Asher L, Fisher R, Zimmerman M (2007). Temporal analysis of Andes virus and Sin Nombre virus infections of Syrian hamsters.. J Virol.

[20] Milazzo ML, Eyzaguirre EJ, Molina CP, Fulhorst C (2002). Maporal viral infection in the Syrian golden hamsters: a model of hantavirus pulmonary syndrome.. J Infect Dis.

[21] Eyzaguirre EJ, Milazzo ML, Koster FT, Fulhorts C (2008). Choclo virus infection in the Syrian golden hamster.. Am J Trop Med Hyg.

[22] Safronetz D, Hegde NR, Ebihara H, Denton M, Kobinger GP (2009). Adenovirus vectors expressing hantavirus proteins protect hamsters against lethal challenge with Andes virus.. J Virol.

[23] Zivcec M, Safronetz D, Haddock E, Feldmann H, Ebihara H (2011). Validation of assays to monitor immune responses in Syrian hamsters (*Mesocricetus auratus*).. J Immunol Method.

[24] Gowen BB, Julander JG, London NR, Wong M-H, Larson D (2010). Assessing changes in vacular permeability in a hamster model of viral hemorrhagic fever.. Virology J.

[25] Zaki SR, Greer PW, Coffield LM, Goldsmith CS, Nolte KB (1995). Hantavirus pulmonary syndrome. Pathogenesis of an emerging infectious disease.. Am J Pathol.

[26] Toro J, Vega JD, Khan AS, Mills JN, Padula P (1998). An outbreak of hantavirus pulmonary syndrome, Chile, 1997.. Emerg Infect Dis.

[27] Rowe RK, Pekosz A (2006). Bidirectional secreation and nonciliated cell tropism following andes virus infection of primary airway epithelial cell cultures.. J Virol.

[28] Padula PJ, Edelstein A, Miquel SD, Lopez NM, Rossi CM (1998). Hantavirus pulmonary syndrome outbreak in Argentina: molecular evidence for person-to-person transmission of Andes virus.. Virol.

[29] Martinez VP, Bellomo C, San Juan J, Pinna D, Forlenza R (2005). Person-to-person transmission of Andes virus.. Emerg Infect Dis.

[30] Castillo C, Naranjo J, Seoulveda A, Ossa G, Levy H (2001). Hantavirus pulmonary syndrome due to Andes virus in Temuco, Chile: clinical experience with 16 adults.. Chest.

[31] Shrivastava-Ranjan P, Rollin P, Spiropoulou CF (2010). Andes virus disrupts the endothelial cell barrier by induction of vascular endothelial growth factor and downregulation of VE-Cadherin.. J Virol.

[32] Rouse BT, Horohov DW (1986). Immunosuppression in viral infections.. Rev Infect Dis.

[33] Tortorella D, Gewurz BE, Furman MH, Schist DJ, Ploegh HL (2000). Viral subversion of the immune system.. Annu Rev Immunol.

[34] Stoltz M, Ahlm C, Lundkvist A, Klingstrom J (2007). Lambda interferon (IFN-lambda) in serum is decreased in hantavirus-infected patients, and in vitro-established infection is insensitive to treatment with all IFNs and inhibits IFN-gamma-induced nitric oxide production.. J Virol.

[35] Robertson SJ, Hasenkrug KJ (2006). The role of virus-induced regulatory T cells in immunopathology.. Springer Semin Immunopathol.

[36] Rouse BT, Suvas S (2004). Regulatory cells and infectious agents: detentes cordiale and contraire.. J Immunol.

[37] Schountz T, Prescott J, Cogswell AC, Oko L, Mirowsky-Garcia K (2007). Regulatory T cell-like responses in deer mice persistently infected with Sin Nombre virus.. Proc Natl Acad Sci U S A.

[38] Easterbrook JD, Zink MC, Klein SL (2007). Regulatory T cells enhance persistence of the zoonotic pathogen Seoul virus in its reservoir host.. Proc Natl Acad Sci U S A.

[39] Easterbrook JD, Klein SL (2008). Immunological mechanisms mediating hantavirus persistence in rodent reservoirs.. PLoS Pathog.

[40] Zhu LY, Chi LJ, Wang X, Zhou H (2009). Reduced circulating CD4+CD25+ cell populations in haemorrhagic fever with renal syndrome.. Clin Exp Immunol.

[41] Saggioro FP, Rossi MA, Duarte MIS, Martin CCS, Alves VAF (2007). Hantavirus infection induces a typical myocarditis that may be responsible for myocardial depression and shock in hantavirus pulmonary syndrome.. J Infect Dis.

[42] Khaiboullina SF, Netski DM, Krumpe P, St Joer SC (2000). Effects of tumor necrosis factor alpha on sin nombre virus infection in vitro.. J Virol.

[43] Mackow ER, Gavrilovskaya IN (2009). Hantavirus regulation of endothelial cell functions.. Thromb Haemost.

[44] Janssen SP, Gayan-Ramirez G, Van den Bergh A, Herijgers P, Maes K (2005). Interleukin-6 causes myocardial failure and skeletal muscle atrophy in rats.. Circulation.

[45] Hedayat M, Mahmoudi MJ, Rose NR, Rezaei N (2010). Proinflammatory cytokines in heart failure: double-edged swords.. Heart Fail Rev.

[46] Afanayeva M, Wang Y, Kaya Z, Stafford EA, Dohmen KM (2001). Interleukin-12 receptor /STAT4 signaling is required for the development of autoimmune myocarditis in mice by an interferon-gamma-independent pathway.. Circulation.

[47] Harrington LE, Hatton RD, Mangan PR, Turner H, Murphy TL (2005). Interleukin 17-producing CD4+ effector cells develop via a lineage distinct from T helper type 1 and 2 lineages.. Nat Immunol.

[48] Tesmer LA, Lundy SK, Sarkar S, Fox DA (2008). Th17 cells in human disease.. Immunol Rev.

[49] Cheng X, Yu X, Ding YJ, Fu QQ, Xie JJ (2008). Th17/Treg imbalance in patients with acute coronary syndrome.. Clin Immunol.

[50] Tang TT, Ding YJ, Liao YH, Yu X, Xiao H (2010). Defective circulating CD4+CD25+Foxp3+CD127^low^ regulatory T-cells in patients with chronic heart failure.. Cell Physiol Biochem.

[51] Hammerbeck CD, Hooper JW (2011). T cells are not required for pathogenesis in the Syrian hamster model of hantavirus pulmonary syndrome.. J Virol.

[52] Terajima M, Ennis FA (2011). T cells and pathogenesis of hantavirus cardiopulmonary syndrome and hemorrhagic fever with renal syndrome.. Viruses.

[53] Sundstrom JB, McMullan LK, Spiropoulou CF, Hooper WC, Ansari AA (2001). Hanyavirus infection induces the expression of RANTES and IP-10 without causing increased permeability in human lung microvascular endothelial cells.. J Virol.

[54] Meissner JD, Rowe JE, Borucki MK, Joer SC St (2002). Complete nucleotide sequence of a Chilean hantavirus.. Virus Res.

