# Supplementary figures and images for: Pathogenesis and Host Response in Syrian Hamsters following Intranasal Infection with Andes Virus
Source: PLoS Pathog. 2011 Dec 15;7(12):e1002426. doi: 10.1371/journal.ppat.1002426 (PMC3240607; doi:10.1371/journal.ppat.1002426)

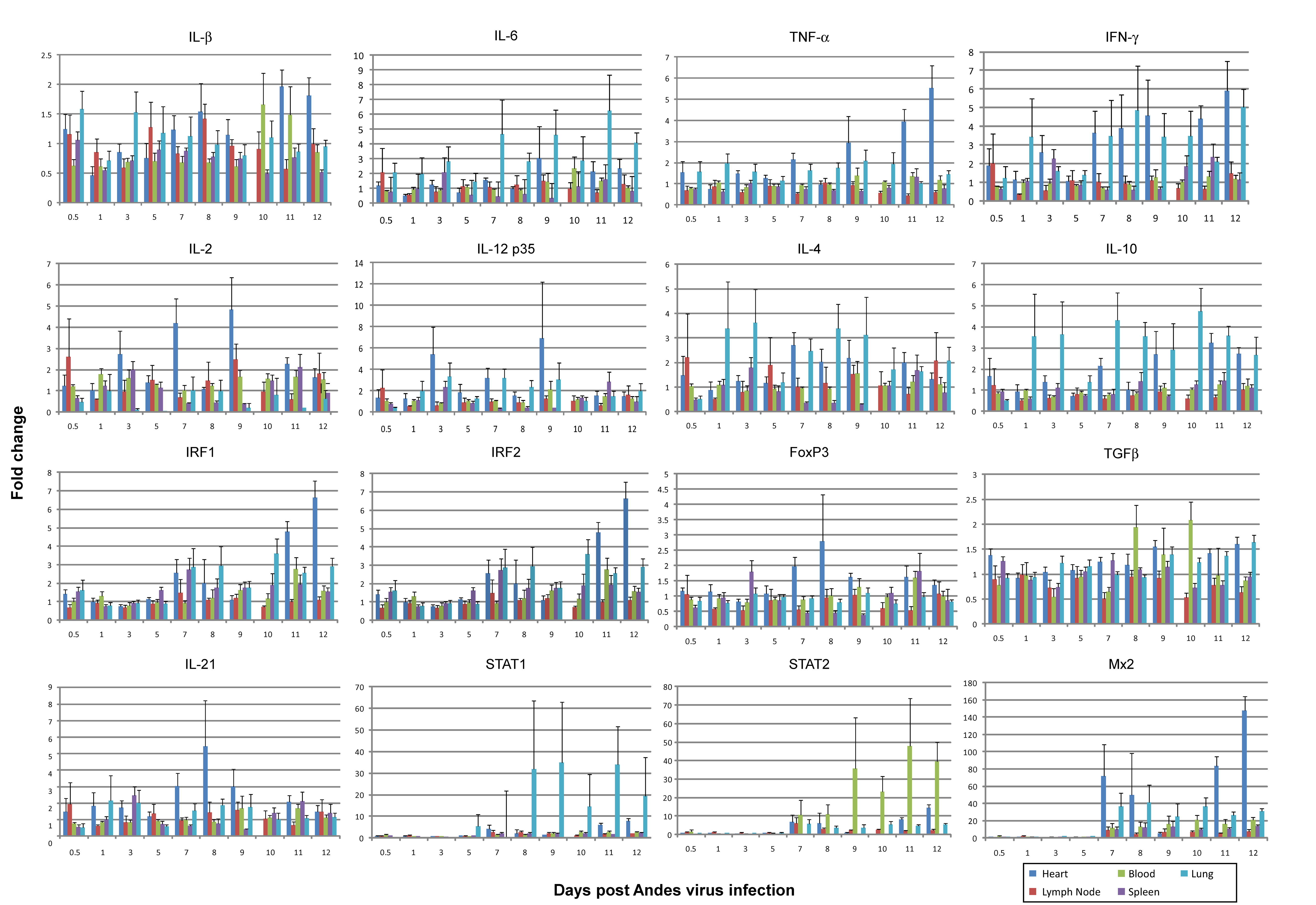

Supplement: Figure S1 — Host responses to Andes virus infection. Host responses to Andes virus infection were monitored using recently developed, hamster specific, real-time RT-PCR assays. Shown is the qRT-PCR data used to generate the heat maps in Figure 7. Error bars represent the standard error of the mean. (TIFF) [file ppat.1002426.s001.tiff]
